# Carbon-Based Materials for Humidity Sensing: A Short Review

**DOI:** 10.3390/mi10040232

**Published:** 2019-03-31

**Authors:** Jean-Marc Tulliani, Barbara Inserra, Daniele Ziegler

**Affiliations:** 1 Department of Applied Science and Technology (DISAT), Politecnico di Torino, C.so Duca degli Abruzzi, 24-10129 Torino, Italy; barbara.inserra@polito.it (B.I.); daniele.ziegler@polito.it (D.Z.); 2 INSTM Research Unit PoliTO, LINCE Laboratory, C.so Duca degli Abruzzi, 24-10129 Torino, Italy

**Keywords:** humidity sensor, carbon-based materials, carbon nanotubes, graphene, carbon black, carbon fibers, carbon soot, biochar, flexible electronics

## Abstract

Humidity sensors are widespread in many industrial applications, ranging from environmental and meteorological monitoring, soil water content determination in agriculture, air conditioning systems, food quality monitoring, and medical equipment to many other fields. Thus, an accurate and reliable measurement of water content in different environments and materials is of paramount importance. Due to their rich surface chemistry and structure designability, carbon materials have become interesting in humidity sensing. In addition, they can be easily miniaturized and applied in flexible electronics. Therefore, this short review aims at providing a survey of recent research dealing with carbonaceous materials used as capacitive and resistive humidity sensors. This work collects some successful examples of devices based on carbon nanotubes, graphene, carbon black, carbon fibers, carbon soot, and more recently, biochar produced from agricultural wastes. The pros and cons of the different sensors are also discussed in the present review.

## 1. Introduction

**Figure 1:**
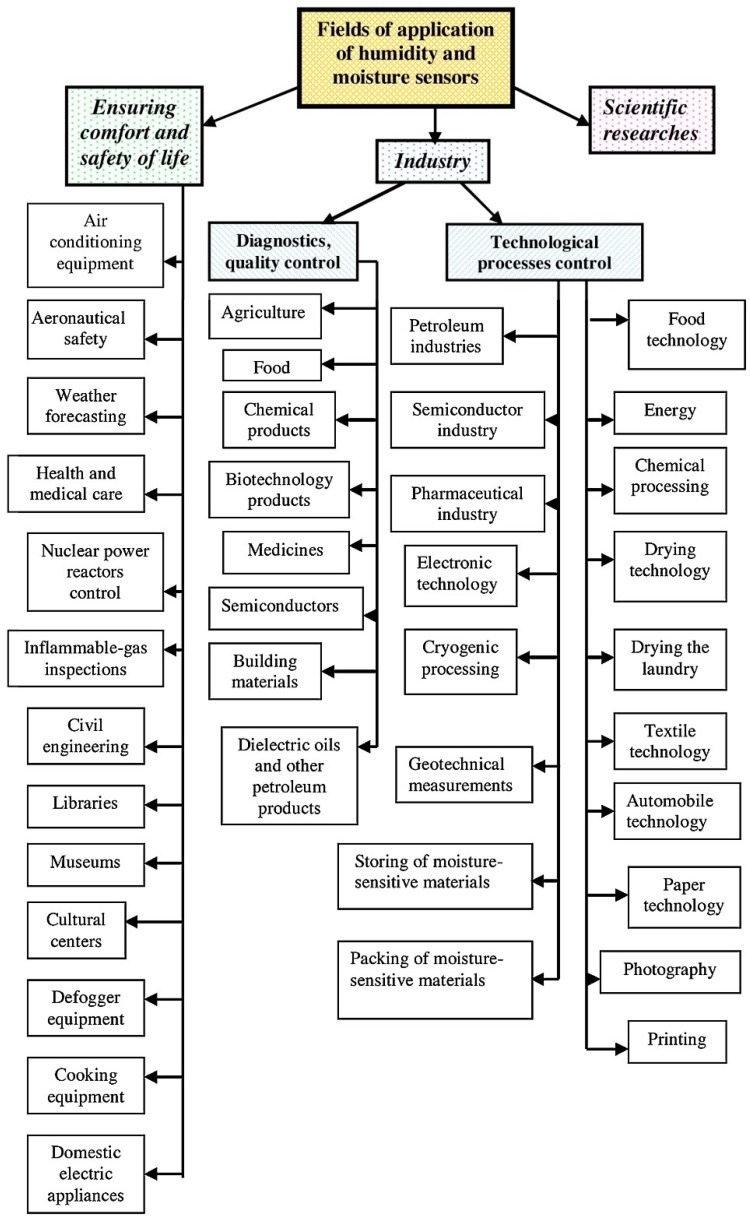
Fields of application of humidity and moisture sensors. Reproduced with permission from [1], published by Elsevier, 2016.

Gas sensors are miniaturized analytical devices that can deliver real-time and on-line information on the presence of a target gas. Humidity sensors are largely used in many fields where accurate and reliable measurements of water content in different environments and materials are of paramount importance, as depicted in Figure 1. These sensors can also constitute a cheap alternative to a laboratory’s analytical technique, so moisture (the water content of any material) sensors and humidity (the water vapor content in gases) sensors are used in many different areas of human activity, such as food quality monitoring, conditioning systems, meteorology, agriculture, manufacturing and process control, medical equipment, and so forth [1]. When air is fully saturated with water, the pressure exerted by the contained water vapor is defined as the saturation water vapor pressure (Ps) that is dependent on the temperature. Thus, the ratio of the current water vapor pressure to the saturation water vapor pressure at a specific temperature is a common way to quantify the amount of water vapor contained in the air [1], and it represents the relative humidity (RH). 

**Figure 2:**
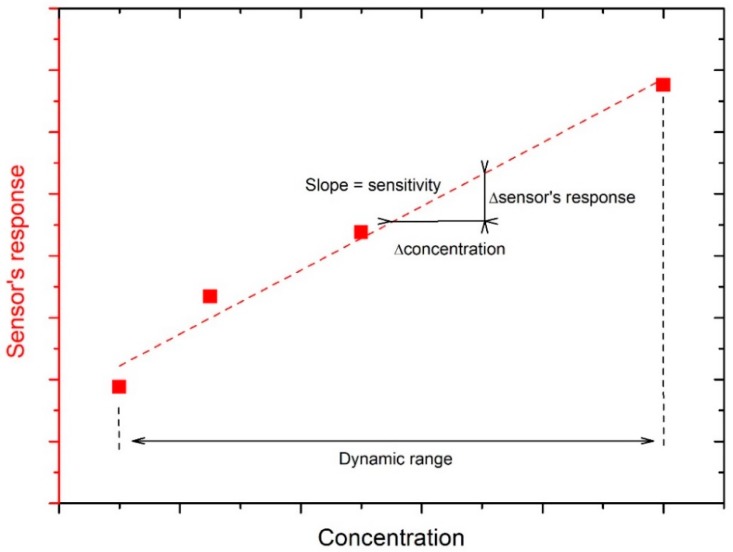
Calibration curve of a sensor exposed to increasing concentrations of an analyte. Elaboration from [2], published by ACS Publishing, 2019.

**Figure 3:**
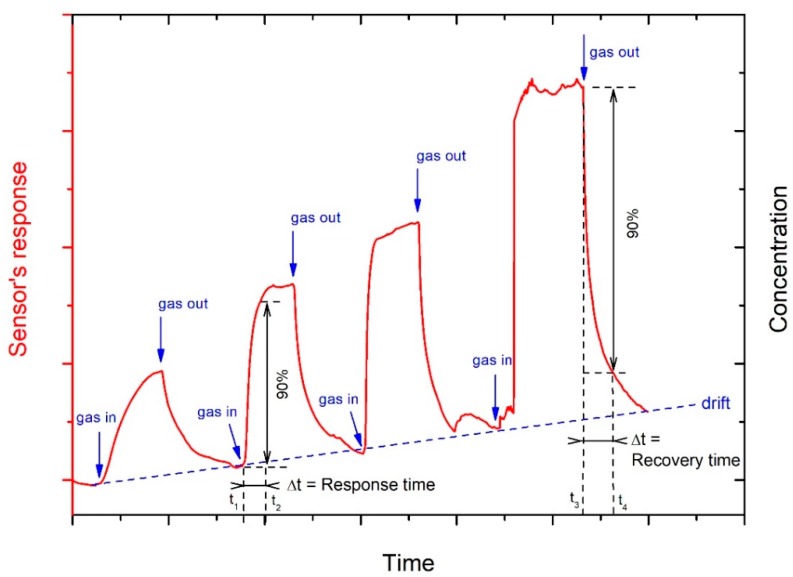
Sensor’s response of a sensor exposed to increasing concentrations of an analyte. Elaboration from [2], published by ACS Publishing, 2019.

The sensor’s response is often determined as the relative changes in a measured physical parameter like the impedance (*Z*), resistance (*R*), current (*I*), conductance (*G* = $\frac{I}{V}$), capacitance (*C*), power gain, or resonant frequency (*f*_0_) value of the device with respect to time. Different conventions have been adopted for plotting these measurements: $\frac{\Delta X}{X_{0}}$, $\frac{X}{X_{0}}$, or simply *ΔX* (where *X* = *Z*, *R*, *I*, *G*, *C*, *f*_0_ or power gain) [2]. Materials that show a resistivity decrease are classified as *n*-type semiconductors, while *p*-type ones are those presenting an increase of the resistivity when the target reducing gas concentration rises. In fact, humidity usually exhibits reducing characteristics, even if the oxidizing effect of water at 300 °C was studied in a recent paper by Staerz et al. on the WO_3_ surface [3]. The limit of detection (LOD) is the lowest amount of target gas that can be detected at a known confidence level [2]. The calibration curve in Figure 2 shows the relationship between the sensor’s response and the concentration of the target gas. The sensitivity of the sensor is given by the slope of the calibration curve (Figure 2). The drift is defined as the change in the sensor’s response over time, independently of the gas concentration (Figure 3). In addition, the selectivity is the ability of the sensor to discriminate the target gas among other species [2].

The requirements for effective humidity sensors that change impedance after exposure to humidity in view of practical applications are the following: good sensitivity (i.e., the capability to discriminate small differences in concentration of the analyte over a wide range of RH values, Figure 2), a short response time (the time that a sensor needs to reach usually 90% of the total impedance or capacitance change during adsorption of gas) and recovery time (the time taken for a sensor to achieve usually 90% of the total impedance or capacitance change in the case of gas desorption) (Figure 3), good reproducibility (small standard deviation in sensor response of devices realized with the same material), great repeatability (small standard deviation in sensor response of the same device under a definite humidity concentration), very small hysteresis (the difference between the impedance value during adsorption and desorption cycles for the same RH value), negligible temperature dependence, low cost of fabrication and maintenance, resistance to contaminants, a linear response, an easy fabrication process, and durability [1]. Finally, a low weight and compatibility with a microprocessor are also required features for some specific applications (e.g., portable devices) [1].

Commercial sensors are mostly based on metal oxides, porous silicon, and polymers, on which water vapor molecules adsorption drastically changes the electrical properties, such as the resistivity and capacity, of the device [4]. Capacitive humidity sensors detect humidity due to a change of capacitance between two detection electrodes. In these devices, the capacitance increases with RH because of a change in the dielectric constant of the sensing material. These sensors exhibit a low power consumption, but have to be calibrated often, according to changes in the sensor permittivity. The resistance or the impedance of the resistive-type sensor decreases as the relative humidity increases for *n*-type semiconductors. Ions or electrons, or both, are the conduction carriers for resistive-type humidity sensors [1,5]. Generally, both resistive and capacitive sensors are cheap and respond over a wide humidity range with good repeatability. However, their response is dependent on the temperature and is influenced by the presence of some other chemical species (i.e., presents low selectivity) [1]. The common construction of the resistive-type ceramic humidity sensors consists of a ceramic substrate with screen-printed interdigitated noble metal electrodes coated with the humidity sensitive materials and a heater to ensure that there is a working temperature in the active region [1]. Currently, this configuration is also the most common for capacitive-type humidity sensors [1]. 

Nowadays, the capacitive-based sensors are dominating the humidity sensor market, with nearly 75% of the sales [6].

Semiconductor metal oxides (SMOs) are sensitive towards different gases. Their working principle is based on the variation of their electrical properties: on the surface of the grains of an *n*-type SMO gas sensor, oxygen molecules can adsorb. These adsorbed oxygen molecules then attract electrons from the conduction band and trap them at the surface as ions, leading to band bending. Therefore, an electron-depleted layer is formed (also known as a space-charge layer). The space-charge region is more resistive than the bulk of the SMO because of electron depletion. It is now widely accepted that below 150 °C, oxygen is adsorbed in ionic form (ionosorbed) as $O_{2}^{-}$ and it dissociates as O^–^ in the temperature range of 150 °C to 400 °C. Above 400 °C, O^2–^ ions are formed [7,8,9,10,11]. In the *p*-type semiconductors, holes are responsible for the conduction and the signal is the opposite compared to *n*-type materials: after exposure to oxygen, a hole-accumulation layer is formed, and the space-charge region is more conductive than the bulk. This causes a drop in the resistance and impedance of the sensitive material.

Optical-type humidity sensors based on change of the optical properties (such as reflectance, evanescent wave, etc.) and acoustic-type humidity sensors based on the variation in the frequency of acoustic resonance both have good sensitivity and acceptable response times [12]. However, the latter two types of sensors cannot be used as flexible sensors due to the complicated measurement systems and non-flexible active materials [12].

The active materials are the most important part in high-performance flexible sensors and should be both flexible and electrically conductive. To this aim, recently, carbon films have attracted great attention for their potential applications as humidity sensors because of their large sensing area and high chemical inertness [4,12]. Due to their rich surface chemistry and structure designability, carbon materials have become interesting in humidity sensing. In addition, carbon nanomaterials, including carbon nanotubes (CNTs), graphene, carbon black, and carbon nanofibers, are among the most commonly used active materials for the fabrication of high-performance flexible sensors. Specifically, CNTs and graphene can be assembled into one-dimensional fibers, two-dimensional films, and three-dimensional architectures, allowing an easy design of flexible sensors for many practical applications [12]. Other advantages of these materials are their ability to work at room temperature (RT), the possibility to functionalize them for chemical specificity, and their low thermal mass that allows rapid heating with low power consumption [13]. Possible drawbacks are their low selectivity, poor reproducibility, tendency to poisoning, and possible long-term drift [13].

Moreover, low-cost carbon materials, including carbon black and carbon nanofibers, can be utilized as sensing materials integrated with fabrics [12]. Besides carbon nanomaterials, other carbon powders derived from bio-materials through pyrolysis have also been proposed as sensing materials, such as silk [14] and cotton [15]. Biomass is also a qualified carbon source, is available at a high quality and huge amount, and is considered an environmental-friendly renewable resource [16]. Furthermore, different biochars, which are residues of biomass pyrolysis, are now available from pilot plants producing biogas and energy [17,18]. In recent years, biochar applications have been conducted in many fields [19], even though the main application of this material remains field amendment in agriculture [20]. Moreover, in recent years, biochar has been extensively studied as a substitution for more expensive materials, such as carbon nanotubes, graphene, and others [21].

This paper reviews different experimental activities dealing with carbonaceous materials used as capacitive or resistive humidity sensing materials with a special focus on biochar. It discusses their advantages and underlines some limits and drawbacks, suggesting useful strategies for minimizing them.

## 2. Sensing Materials

### 2.1. Carbon Nanotubes (CNTs)

In the last two decades, various resistive-type humidity sensors based on polymers (polyimide) [22], sulfonated polyimides (SPIs) [23], poly(2-acrylamido-2-methylpropane sulfonic acid) [24], poly(2-acrylamido-2-methylpropane sulfonate) [25], and poly(4-vinylpyridine)/poly(glycidyl methacrylate [26]) and/or carbon nanotubes [27] have been investigated. Polymer-based resistance sensors are limited in their ability to detect low levels of humidity, mostly because of their high initial resistance value: the minimum detectable RH concentration is around 42% for a polyimide-based resistive humidity sensor [22] and is equal to 30% for a sulfonated polyimide [23]. Thus, more sensitive CNTs-based sensors were developed [5]. 

CNTs are excellent sensor candidates due to their mechanical and electrical properties, as well as their ability to be functionalized and their easy integration into electronic circuits [28]. CNTs can be either single-walled (SWCNTs) or multi-walled (MWCNTs). MWCNTs are made of multiple concentric layers of SWCNTs. In defect-free tubes, the bonds between carbon atoms in sidewalls are hybridized sp^2^ and noncovalent van der Waals forces or π stacking dominate the intermolecular interactions [28]. MWCNTs contain both holes and electrons and at room temperature, present a metallic behavior because of the overlapping of conduction and valence bands with electrons as majority carriers [27,29,30]. MWCNTs can also behave as semiconductors with the energy overlap changing in function of the chirality and thus, the interaction between the different MWCNTs walls [31,32].

In Varghese et al. [27], MWCNTs were grown by the pyrolysis of ferrocene and xylene under an Ar/10% H_2_ atmosphere in a two-stage reactor. Ferrocene acted as a Fe catalyst and xylene as a carbon source. The liquid was first pre-heated at 175 °C and subsequently sent to the reactor at 750 °C. MWCNTs were deposited on quartz substrates with interdigitated electrodes to keep the impedance of the sensor low, while providing a maximum surface area for contact with the gas atmosphere. MWCNTs showed a considerable response to humidity with a response time of 2–3 min when increasing RH values, while the recovery times were in the order of few hours [29]. The results revealed a charge transfer between the water molecules and the MWCNTs, typical of a dominant chemisorption process since physisorption does not involve any charge transfer [29]. For *p*-type MWCNTs, the adsorbed water molecules extract holes to the valence band, leading to an increase in the resistance of the film. The proposed sensors showed a cut-off (i.e., sensors start to respond) from 10 RH% and 20 RH%, respectively, for the capacitive and resistive sensors [29].

In Lee et al. [5], poly (acrylic acid) (PAA) was used to disperse the MWCNTs, as PAA is highly hygroscopic and sensitive to low RH levels. To disperse high amounts of MWCNTs in PAA, poly (4-styrenesulfonic acid) (PSS) was used as a surfactant as PSS wraps the MWCNTs’ hydrophobic outer shells with noncovalent polymer chains [5]. The PSS and MWCNTs were first mixed together in a mortar to let the MWCNTs wrap with the PSS surfactant. The PSS/MWCNT mixtures were then wetted with distilled water and ground for 10 min to prevent aggregation of the entangled MWCNTs. The mixtures were diluted in distilled water to disperse the nanotubes, prior to sonication for 15 min in an ultrasonic bath, followed by sonication for other 15 min with an ultrasonic probe. Subsequently, PAA powder was dissolved in the MWCNT mixtures at 85 °C by stirring for 1 day. Finally, the sensor was manufactured by depositing a 20 μL drop of this solution on a polyimide film with gold interdigitated electrodes. The MWCNT/PAA films contained up to 33 wt% of MWCNTs [5]. The humidity sensor exploits the volume’s change of the MWCNT/PAA film due to humidity adsorption and desorption, which causes an increase or a decrease in the distance between neighboring MWCNTs. Thus, the electrical resistance of the film is dependent on volume changes caused by the adsorption and desorption of water vapor. Films with more space to absorb humidity show a higher degree of swelling, especially with a higher content of PAA. The film made with the ratio of MWCNTs/PAA equal to 1:4 exhibited good sensitivity to humidity: its resistance variation from 30 RH% to 90 RH% was equal to 930 Ω. This result was due to the high concentrations of MWCNTs in the polymer matrices, which led to limited changes in resistance with increasing RH levels. However, the response to humidity was highly linear and the sensitivity was acceptable [5]. The response and recovery times were equal to 670 s and 380 s, respectively (for RH levels increasing from 50% to 90% and decreasing from 90% to 50%). 

**Figure 4:**
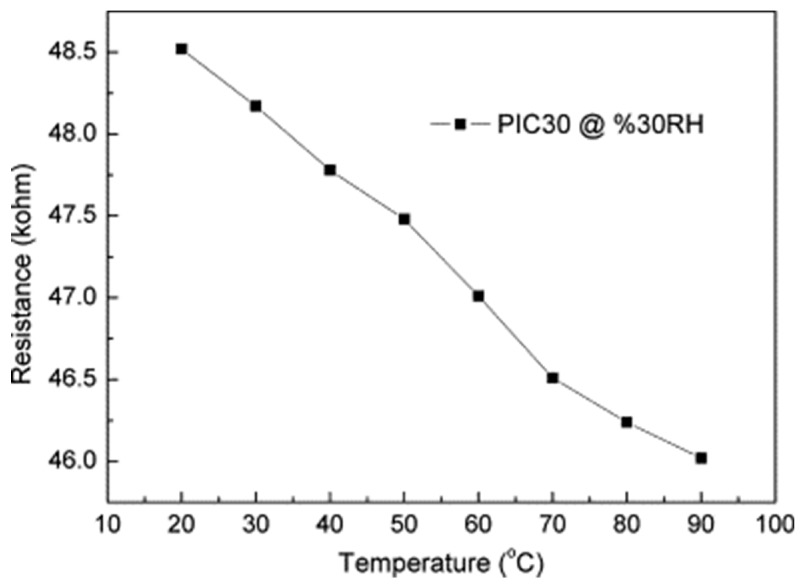
Resistance changes in function of temperature for PIC30 sensor. Reproduced with permission from [32], published by Elsevier, 2011.

Polyimide (PI)/MWCNTs composite films were prepared by in-situ polymerization at room temperature in Tang et al. [32]: the precursors (polyamic acid, PA) were obtained by stirring a mixture of pyromellitic dianhydride (PMDA), 4,4-oxydianilline (ODA), and surfactant-dispersed MWCNTs in N,N-dimethylacetamide (DMAc) for 4 h. A series of PA/MWCNTs nanocomposites (with theoretical contents of MWCNTs in the PI of 0.5, 1.0, 2.0, and 3.0 wt% and labeled respectively as PIC05, PIC10, PIC20, and PIC30) were synthesized with the same PA solid content of 18%. The precursors were then coated on glass substrates and kept in an oven at 60 °C for 12 h to let the solvent evaporate. Subsequently, step curing at temperatures of 100, 150, 200, 250, and 300 °C for 1 h was used to obtain PI/MWCNTs films. The sensitivity of PIC10, PIC20, and PIC30 at 30 °C was 0.00138/RH%, 0.00178/RH%, and 0.00146/RH%, respectively. The sensitivity of the PIC30 film is lower compared to that of the PIC20 film, and the sensitivity of composites is higher just around the percolation threshold. In PIC films, the percolation threshold is around 1 wt%. A higher content of MWCNTs make the carbon nanotubes tightly connected to each other and create physical contacts in the network. Therefore, the response due to polymer swelling is smaller compared with the sample near the percolation threshold. Finally, the resistance of the PIC30 film decreased when the temperature increased (Figure 4); thus, temperature compensation should be made when this sensor is used at different temperatures. This negative temperature dependence indicates that the major conductive mechanism of PIC30 was due to the inter-tube effect: higher temperatures provided more energy for the motion of electrons lowering the barriers between MWCNTs, resulting in a decrease of the film resistance.

In Yoo et al. [33], pristine and radio frequency oxygen plasma-treated MWCNTs (p-MWCNTs) were mixed with PI and spin-coated on a Si_3_N_4_ membrane. Their resistance values continuously rose with the increasing RH levels, in the relative humidity range 10%–90%, while the initial resistance value decreased with the increasing CNTs content. The measured sensitivities are reported in Table 1.

**Figure 5:**
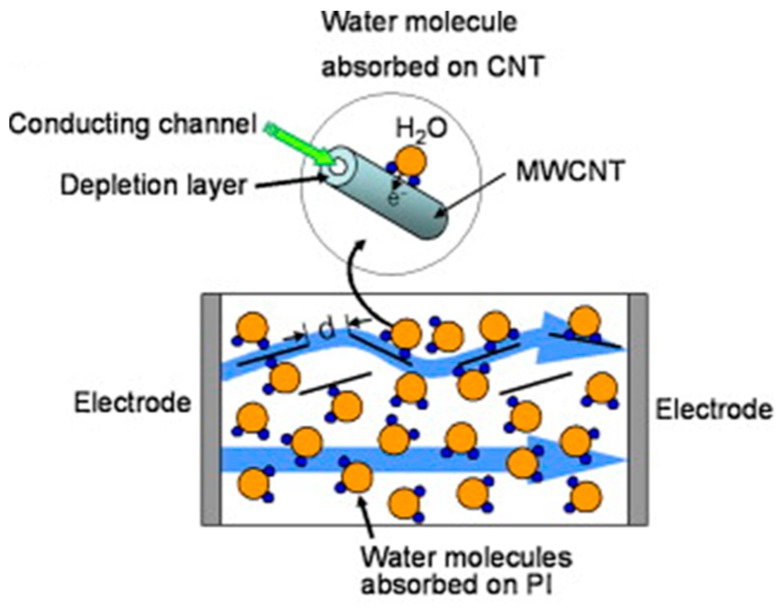
Scheme of the conduction mechanism of *p*-MWCNT/PI composite devices. Reproduced with permission from [33], published by Elsevier, 2010.

The p-MWCNTs/PI sensors showed better linearity and sensitivity than the non-treated ones and a scheme of the gas sensing mechanism is depicted in Figure 5.

The authors explained this result by stating that the surface of the plasma-treated CNTs has a higher presence of defects with respect to pristine ones. They proposed electrical conduction as a bulk phenomenon in contrast to surface adsorption. H_2_O molecules in PI possess a polarizability that is around 80% with respect to the condition of free water. As a result, water species are not significantly involved in hydrogen bonding and are situated primarily in free volumes in the polyimide network. Consequently, the high conductivity water in the PI network increases the conductivity of the PI-H_2_O system. 

Water molecules can be either chemically bound (with the oxygen of the ether linkage and with the four carbonyl groups at low RH levels) in the PI network or can condense in microvoids at higher humidity amounts [34]. As previously described, only some of the water molecules in PI composites films can bind to the CNTs through the hydrogen atom [33]. When the MWCNTs concentration is much lower than the percolation threshold, the distance between the nanotubes is large and CNTs are almost isolated. Thus, the resistance of the sensor is governed by the resistance of the water adsorbed on PI and the sensor’s response is nonlinear. On the contrary, when the MWCNTs concentration is close to the percolation threshold, tunneling through a potential barrier across the MWCNTs is effective. In the *p*-MWCNTs/PI sensors, the intertube’s resistance increases due to the adsorption of water molecules, while the PI resistance decreases, leading to an almost flat sensor’s response in the investigated relative humidity range. If the *p*-MWCNTs fraction is above the percolation threshold, the number of CNT-CNT connections rises and more current paths through intertubes are effective. Then, the adsorption of water molecules on CNTs becomes important. The amplified sensor’s resistance is due to the transfer of electrons from H_2_O molecules to carbon nanotubes and/or the intertube’s distance variation when PIs begin to swell. The electron transfer caused by the adsorption of water molecules will shift the valence band of the CNTs away from the Fermi level, reducing the hole concentrations and increasing the depletion layer thickness. Thus, the resistance of the MWCNTs and of the intertube tunneling barrier will increase [33]. 

Resistive-type humidity sensors made from composite films of hydroxyethyl cellulose (HEC) and MWCNTs (purity > 99.5%; 7, 8 and 9 wt%) were investigated in Ma et al. [35]. The sensors were prepared by dissolving 0.3 g of HEC powder with MWCNTs in 3.0 g of distilled water at 80 °C for 5 min. Then, the solution was centrifuged for 10 min and defoamed for 1 min. The final mixture was pasted between two electrodes with wires onto an alumina plate. The ceramic plate was finally dried at 130 °C for 1 h to remove the distilled water. The sensor’s response (Δ*R*/*R*_0_) varied in an exponential form. When the sensor’s response was expressed on a logarithmic scale, the sensitivity of the studied sensors was 0.0866/RH%, 0.0937/RH%, and 0.10069/RH%, respectively, for the 7 wt%, 8 wt%, and 9 wt% MWCNTs films. The HEC/MWCNTs sensor with 7 wt% MWCNTs showed the best performance from a repeatability and stability point of view, in the dry/wet cyclic tests. However, the HEC/MWCNTs sensor with 9 wt% MWCNTs presented the highest sensitivity. 

**Figure 6:**
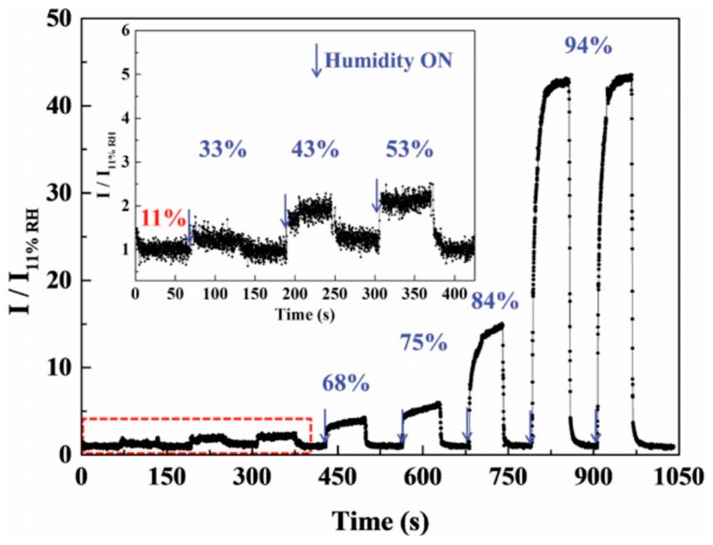
Dynamic response curve for the MWCNTs/PVP film sensor heat-treated at 350 °C at different RH% values. Reproduced with permission from [36], published by Wiley Online Library, 2016.

In the study of Pan et al. [36], MWCNTs were first functionalized by chemical treatment: 150 mg of MWCNTs was ultrasonicated for 4 h in a mixture of concentrated H_2_SO_4_/HNO_3_ at a ratio of 3:1. The treated MWCNTs were then filtered over a 0.45 μm pore size membrane and the filter cake was washed with deionized water. Subsequently, the remaining solid was suspended in solution of H_2_O_2_ (20 v/v%, 150 mL) in an ultrasonic bath for 2 h. Finally, the product was filtered and dried overnight in a vacuum oven at 50 °C. The oxidized MWCNTs (100 mg) were dispersed in N-N-dimethylformamide (DMF, 8.55 mL) under ultrasonication for a few minutes until a stable suspension was formed. Then, polyvinylpyrrolidone (PVP, 900 mg) was added into the suspension with stirring and the mixture (MWCNTs/PVP weight ratio equal to 1:9) was kept under stirring for another 24 h. The MWCNTs/PVP sensors were prepared on quartz slides with interdigitated electrodes and the films were dried at room temperature for 12 h and at 60 °C for 12 h. Finally, the MWCNTs/PVP films were heat-treated at 350 °C for 1 h. The response was defined as I/I_11%_, where I was the current of the sensor at different RH values and I_11%_ was the current of the sensor at 11 RH%. The films started to respond to humidity from 33 RH% (Figure 6). The response and recovery times between 11 RH% and 94 RH% were about 15 and 1.8 s, respectively [36]. 

The interfacial heterojunctions between MWCNTs and PVP strongly influence the sensitivity and response/recovery times of the sensors. At low relative humidity values, few carriers (H^+^ and H_3_O^+^) were injected into the heterojunctions due to the adsorption of water molecules. The ions carrier injected into *n*-type PVP neutralized the intrinsic electrons of PVP and weakened the barrier of *n*–*p* heterojunctions, decreasing the resistance of heterojunctions. Therefore, the MWCNTs/PVP sensor exhibited a good sensitivity at low RH. At high relative humidity values, the swelling of PVP was strongly limited because of the stability of the MWCNTs net. This allowed the MWCNTs/PVP sensors to exhibit excellent repeatability (Figure 6, the two successive measurements at 94 RH%). In addition, with decreasing MWCNTs content, the number of interfacial heterojunctions and sensitivity decreased, and longer response/recovery times compared with the 10% MWCNTs/PVP sensor were determined [36]. 

**Figure 7:**
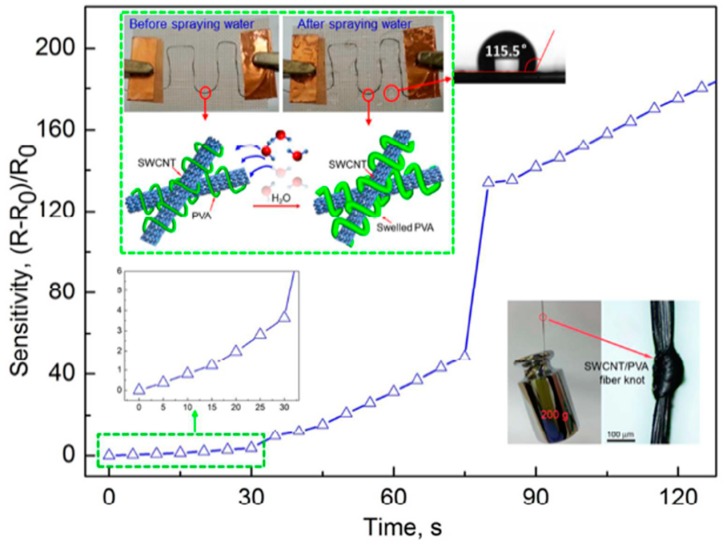
Single-walled carbon nanotube (SWCNT)/Poly (Vinyl Alcohol) (PVA) filament sensor response. Reproduced with permission from [37], published by ACS Publishing, 2017.

The results of a wearable textile-based humidity sensor utilizing high strength (~750 MPa) and ultra-tough SWCNTs/PVA filaments made by a wet-spinning process were reported in [37] (Figure 7). The diameter of an SWCNT/PVA filament under wet conditions was two times that under dry conditions. Moreover, the electrical resistance of a fiber sensor stitched onto a hydrophobic textile significantly increased by more than 220 times after water was sprayed. Textile-based humidity sensors using a weight ratio of 1:5 between SWCNT and PVA filaments showed a great sensor response. In fact, the electrical resistance increased by more than 24 times under 100% of RH with respect to 2.4 times when the molar ratio was 1:1. Moreover, the response time was rather short (40 s). The effect of operating temperature was also investigated, and a significant decrease in the sensor response of around 50% was measured when the operating temperature increased from 25 to 75 °C. The authors have demonstrated that the textile sensor based on the SWCNT/PVA filament can be utilized to monitor human sweating and water leakage on a high hydrophobic textile, with a contact angle of 115.5°. The as-fabricated sensor also displayed an excellent reversibility under high relative humidity values and wet conditions. 

In [38], carbon nanotube (CNT)-yarn humidity sensors were manufactured using an MnO_2_-coated CNT yarn and the sensor performances were compared with those of a pure CNT yarn. The results of this work demonstrated that an increase in humidity causes a decrease in the hole density of *p*-type nanotubes, resulting in an increase in the resistance of the sensors. An Fe film acting as a catalyst for CNTS growth was first deposited by electron beam evaporation on 330-μm-thick *p*-type silicon wafers and then annealed in a vertical cylinder atmospheric-pressure chamber. After purging the tube with He for 10 min, the temperature in the chamber rose to 780 °C in 15 min. The CNTs were grown at 780 °C by adding acetylene gas to the flow for 5 min. Subsequently, acid treatment was carried out in solutions of H_2_SO_4_/HNO_3_ (3:1, 50 mL) for 1 h at room temperature. The aim of the acid treatment of the CNT during functionalization is to introduce hydrophilic carboxylic functional groups to the sidewalls, in order to to improve the performance of the CNT humidity sensor. After functionalization, MnO_2_ nanoparticles were directly electrodeposited on the CNTs in a fresh aqueous solution of 0.02 M of MnSO_4_·H_2_O and 0.2 M Na_2_SO_4_ at pH ~5.6.

**Figure 8:**
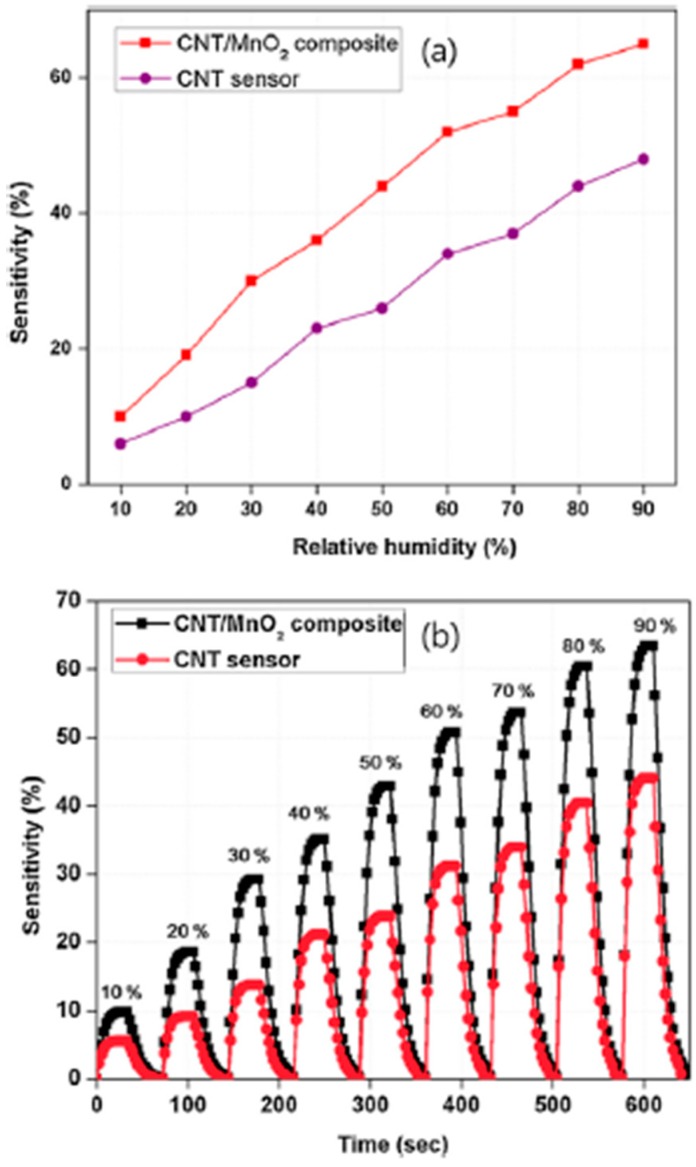
Sensitivities of humidity sensors as a function of relative humidity. (**a**) and as a function of time; (**b**) between 10 and 90% RH. Reproduced with permission from [38], published by Elsevier, 2015.

MnO_2_ was electrodeposited at a potential of 0.9 V for 40 s, and this resulted in a 150 ± 30 nm thick MnO_2_ on CNT yarn. Then, a p-n heterojunction was formed at the interface between the CNT and MnO_2_ nanoparticles, after the electrodeposition of MnO_2_ on the CNT. This produced band bending in the depletion layers. Because of the relative position of the CNT and of the conduction band edges of MnO_2_, the electrons transferred to the conduction band of MnO_2_ can be injected to the CNT. Eventually, this process leads to a decrease in the concentration of holes in the CNTs, giving rise to an increase in the electrical resistance of the CNT/MnO_2_ composite. In this case, MnO_2_ provides active sites for the adsorption of H_2_O molecules and constitutes an excellent medium for electron transfer during the sensing process. In this way, the sensitivity of the CNT/MnO_2_ composite is significantly improved. A comparison of the two sensors’ performances towards humidity is depicted in Figure 8.

### 2.2. Graphene

**Figure 9:**
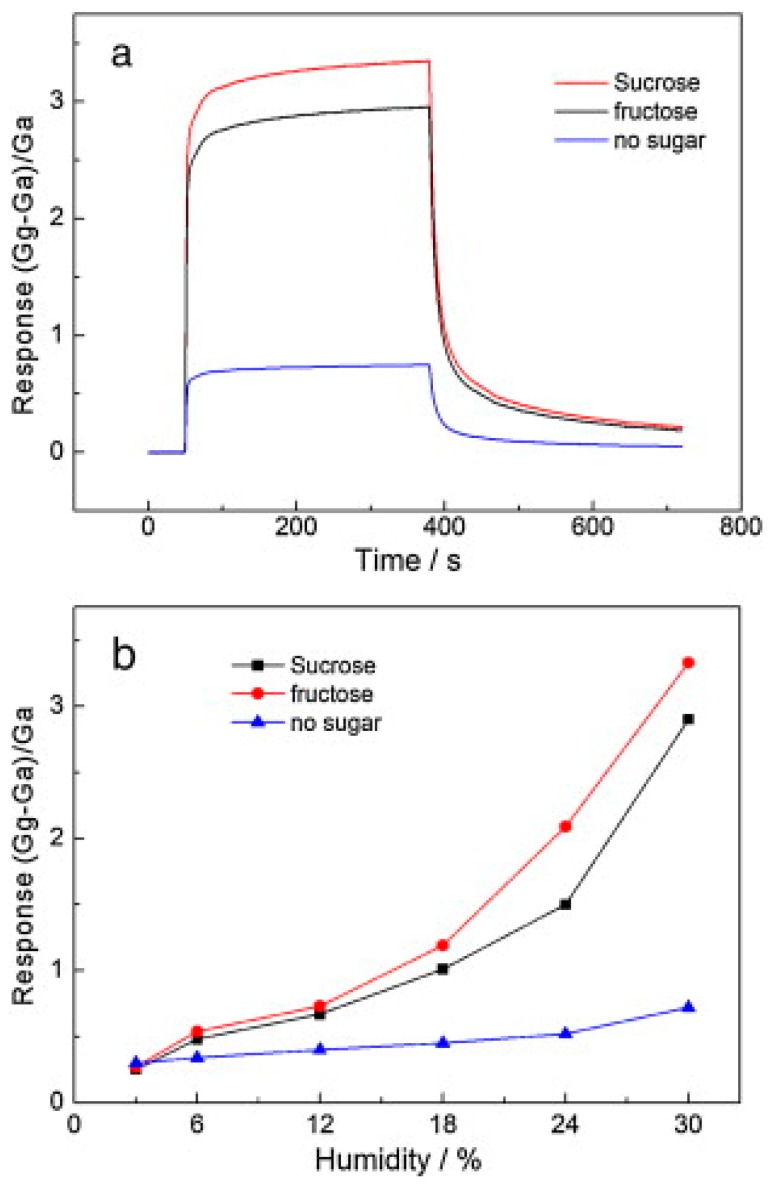
Dynamic response under 30% RH: (**a**) response in terms of conductance’s changes in the range 3–30% RH; (**b**) for different graphene-based sensors. Reproduced with permission from [39], published by Elsevier, 2012.

In the work of Huang et al. [39], sodium (5 g) and sugar (fructose or sucrose, 5 g) first reacted with ethanol as a solvent (50 mL) in a sealed Teflon autoclave at 220 °C for 72 h. The addition of sugar was aimed at creating defects in the graphene sheets. The precursor was then rapidly pyrolyzed at 600 °C for 2 h and washed with deionized water and methanol, prior to filtering and drying. One drop of a graphene methanol solution was subsequently deposited onto an alumina substrate with gold interdigitated electrodes and dried for 5 h to evaporate the solvent. Finally, the device was annealed in vacuum at 180 °C for 2 h to improve the contact. In the absence of any sugar, the response changed slightly from 3 to 30 RH%; however, for the sample with sucrose, the response increased sharply from 0.27 to 3.33 in the same RH range. In addition, the cut-off was around 6 RH% when sugar was used during the synthesis process (Figure 9). XPS, XRD, and Raman spectroscopy results demonstrated that oxygenated groups increased from 17.7% in the sample with no sugar to 24.7% in the sample obtained with sucrose.

**Figure 10:**
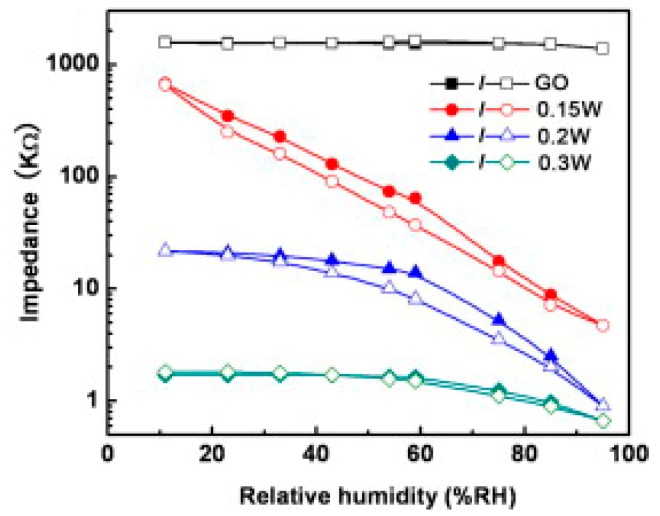
Impedance changes of GO-based sensors. Reproduced with permission from [42], published by Elsevier, 2012.

Unlike graphene and reduced graphene oxide (rGO), graphene oxide (GO), because of its oxygen functional groups, is strongly hydrophilic and proton conductive [40,41]. This makes GO an ideal candidate for humidity detection. The Van der Waals interaction between H_2_O and graphene is weak (0.044 eV) while H_2_O forms hydrogen bonds with the epoxy (0.201 eV) and hydroxyl groups (0.259 eV) [42,43]. Guo et al. [42] used GO produced by Hummers’ method from natural graphite (Aldrich, <150 μm) to prepare sensing films on poly(ethylene terephthalate) (PET) substrates by the spin-coating technique. After drying at 60 °C for 1 h, the samples were exposed for 10 s to two beams which were split from the UV laser to reduce and produce patterned hierarchical nanostructures. The content of oxygen atoms in pristine GO was 46.5%, with only 32% of carbon atoms not bonded to oxygen. After reduction with 0.15 W laser treatment, the C–C percentage increased to 68% and C–O percentage decreased to 23%, indicating the loss of oxygen groups from the surface. An increase of the laser power led to a further reduction of the GO film. After two-beam-laser interference (TBLI) reduction with the laser power of 0.15 W, the resistance of the devices dropped by more than two orders of magnitude when RH increased in the 11%–95% range. Moreover, the sensors’ response showed good linearity and limited hysteresis (6%) under 60 RH% (Figure 10). 

When the laser power was increased to 0.2 W and 0.3 W, the range of the resistance change became smaller and the resistance versus RH% was no longer linear over a wide humidity range (Figure 10). This result was due to the relatively higher conductivity and to the damaging of hierarchical nanostructures compared with the device fabricated with the 0.15 W laser. For the sensor realized with the laser power of 0.15 W, the response time was very fast (2 s) when increasing from 11 to 95 RH%. Then, when decreasing the RH level from 95% to 11%, the recovery time was higher than 100 s. For the device fabricated by the laser power of 0.2 W, the response time was about 3 s, and the recovery time was about 10 s. Finally, when the laser power increased to 0.3 W, the response time increased until 50 s, while the recovery time was about 3 s [42].

GO was obtained from natural graphite powder by an oxidation reaction according to a modified Hummers’ method in Yu et al. [44]. GO ethanol solution (50 mL) with a concentration of 1 mg/mL was sealed in a 100 mL autoclave and then heated to 180 °C for 12 h. After that, the autoclave was left to cool down naturally to room temperature. The prepared ethanol intermediates were removed from the autoclave by a slow and progressive solvent exchange with water, prior to drying with a freeze-dryer and then, at 120 °C for 2 h in a vacuum oven. Subsequently, the sample was annealed at 450 °C in H_2_/Ar (5/95, v/v) for 6 h. Finally, the sample was treated in a UV ozone system for 15 min to obtain the final 3D graphene foam (3DGF). When the RH level rose, the obtained channel currents of the sensor decreased continuously. The response and recovery times were determined for the sensor when the RH level was changed from 0 to 85 RH% and from 85 RH% to 0 RH% and were approximately equal to 89 ms and 189 ms, respectively.

**Figure 11:**
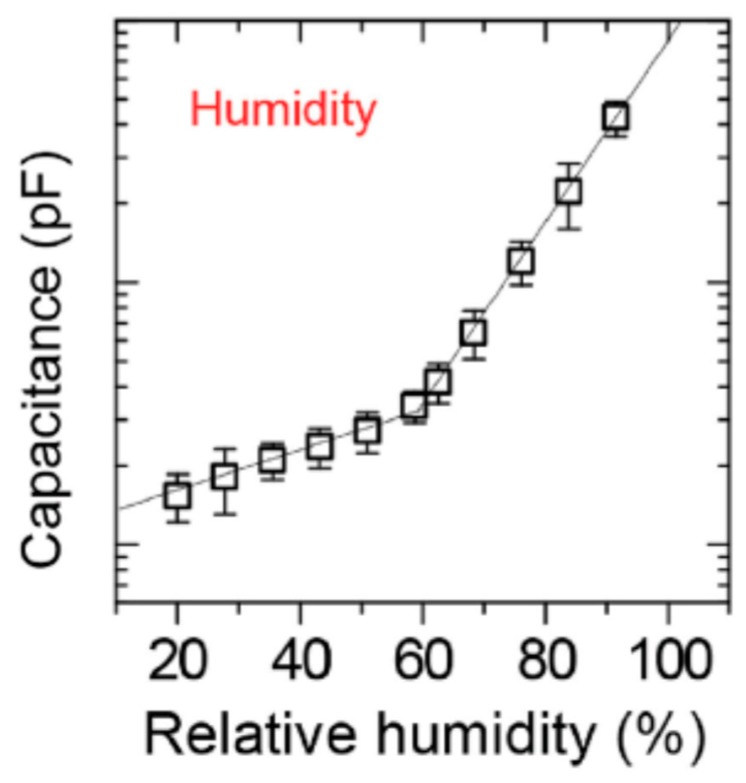
Impedance change of GO-based sensors. Reproduced with permission from [46], published by Wiley Online Library, 2016.

Graphene oxide was prepared from natural graphite (Alfa Aesar, 99.999% purity, 200 mesh) using a modified Hummers’ method [45]: graphite and H_2_SO_4_ were first mixed in a flask and KMnO_4_ was added slowly over 1 h. After 2 h stirring, the solution was kept over an ice water bath. Subsequently, the mixture was stirred vigorously for 18 h and deionized water was added. Then, the solution was stirred for 10 minutes over an ice-water bath and H_2_O_2_ (30 wt% aqueous solution) was added. Finally, the mixture was stirred for 2 h. The resulting mixture was precipitated and filtered to obtain the graphite oxide powder (GO). The GO was then exfoliated into GO nanosheets in water by bath sonication for 1 h. Subsequently, the GO nanosheets were dispersed in N-methyl-2-pyrrolidone to limit aggregation. Hydrazine monohydrate (N_2_H_4_) was then added dropwise to the GO solution to a final concentration of 4 mM. The GO was finally reduced in solution by heating at 100 °C for 24 h. Large-area graphene electrodes were grown on a Cu foil (10 × 10 cm^2^) through CVD. The Cu foil was inserted into a quartz tube and annealed at 1000 °C under an H_2_ atmosphere for 1 h. Then, 5 sccm of CH_4_ were introduced to trigger graphene growth under a continuous H_2_ flow of 10 sccm. After 30 min, the CH_4_ flow was stopped and the tube was cooled down to room temperature under H_2_ flow. The graphene obtained on the Cu foil was then transferred onto a polydimethylsiloxane (PDMS) substrate using standard photolithography and etching processes [45]. The PDMS solution with a base prepolymer and a crosslinking agent (in a weight ratio of 10:1) was poured onto a hexamethyldisilazane-treated glass, and the sample was then put into a chamber to remove bubbles in the PDMS layer. After degassing, the glass with the PDMS layer was spin-coated and cured at 120 °C for 1 h, and then converted into a hydrophilic surface with O_2_ plasma. When the relative humidity was increased from 20% to 90%, the capacitance increased from 0.15 to 4.27 pF because the adsorbed water molecules increased the GO capacitance (Figure 11). Two distinct regimes were evidenced in the curves. For RH levels below 60%, water molecules were adsorbed onto the GO surface through double hydrogen bonding. In this regime, the protons’ hopping between adjacent hydroxyl groups increased the leak conductivity in the film, enhancing the capacitance of the GO film. As the RH rose above 60%, a larger number of water molecules were adsorbed onto the GO surface and penetrated the GO films. These water molecules made the hydrolysis of carboxyl, epoxy, and hydroxyl groups on the GO surface easier. These ionic species sharply enhanced the ionic conductivity and thus the sensor’s capacitance increased exponentially [46].

In Borini et Al. [47], GO humidity and temperature sensors with PEN (polyethylene naphthlate) as supporting material and silver interdigitated electrodes were realized by drop casting and spray coating methods. The manufactured sensors had different thicknesses (15 nm, 25 nm, and 1 µm). Sensors obtained by spray coating, with a GO layer roughly 15 nm thick, showed ultrafast response and recovery time, almost 30 ms, due to the high graphene permeability and to its 2D structure. This result allows classifying the sensor among the fastest ever produced before. The speed of the sensors was studied as a function of the film thickness, response time 20–30 ms and recovery times 90 ms for 25 nm thick films—30 ms for 15 nm thick films were determined. The response of a 25 nm thick film is lower than that of the 15 nm one. The measurements were done in the R.H. range from 30% to 80% and temperature range 10–40 °C.

GO shows great potential in the sensor technology. For example, it can be exploited, beyond RH and T, to monitor the littlest variation of moisture during a vocal expression.

In [48], a layer of graphene was deposited on the surface of a chip made of p-doped silicon substrates with a 300 nm thick SiO_2_ layer by using a CVD graphene wet transfer technique after deposition on a copper foil. The graphene layer was finally patterned using a photoresist mask and O_2_ plasma etching. The sensitivity of the sensor in the investigated RH range was 0.31 1/RH%, with very fast response and recovery times (respectively equal to 0.4 and 0.6 s).

A flexible humidity sensor based on SnO_2_/reduced graphene oxide (RGO) nanocomposite film was proposed in [49]. The humidity sensor was fabricated on a flexible polyimide (PI) substrate with microelectrodes by one-step hydrothermal synthesis. Compared with traditional humidity sensors, the as-prepared sensor demonstrated an ultrahigh sensitivity together with rapid response and recovery characteristics. The electrodes were fabricated by metal sputtering. In the microfabrication of the sensor, a 20 μm thick Cu/Ni layer was firstly deposited on the PI substrate (75 μm thick) with a sputtering system. Subsequently, photoresist (PR) was applied to make a pair of interdigital electrodes (IDEs) pattern with the lithography technique, and subsequently, the redundant Cu/Ni was etched out to form micro-IDEs. A sensing film of an SnO_2_/RGO hybrid composite was finally realized by a hydrothermal treatment with an SnCl_4_ solution in the presence of graphene oxide. Firstly, 2 mL of GO (0.5 mg·mL^−1^) and 24 mg of SnCl_4_·5H_2_O were added into 20 mL of deionized water by sonication for 10 min and stirring for 1 h. The solution was then transferred into a 40 mL Teflon-lined, stainless-steel autoclave and heated at 180 °C for 12 h, during which GO was converted into conductive rGO under hydrothermal reduction. After the autoclave was cooled down, the as-prepared products were centrifugated for 10 min and subsequently washed with deionized water. Finally, the resulting SnO_2_/RGO dispersion was drop-casted onto the flexible substrate, followed by vacuum-drying in an oven at 50 °C for 2 h. The sensor showed a great variation in capacitance from 246.53 pF to 138267 pF over the humidity range from 11% to 97 RH%. The corresponding capacitance changed by approximately 550-folds of magnitude within the entire humidity range of 11–97 RH%. 

A highly sensitive humidity sensor made of silver inter-digital electrodes and a graphene (G)/methyl-red (M-R) composite layer deposited on a low cost transparent polyethyleneterephthalate (PET) substrate through the inkjet printing technique was obtained in [50]. In order to achieve a high sensitivity and a wide sensing range, the methyl-red composite thin film layer was deposited over the silver interdigital electrodes through electrohydrodynamic (EHD) and its thickness was ~300 nm. The graphite powder (0.05 g) was first dispersed in NMP (10 mL) solvent and the solution was then sonicated in an ultrasonic bath for 30 min at room temperature. After bath sonication of the ink, large un-exfoliated graphite flakes were separated by vacuum filtration. Finally, the ink was centrifugated for 30 min and the supernatant was separated from sediment. 10 wt% methyl-red was prepared in dimethylformamide (DMF) by bath sonication for 2 h at 30°C. This temperature was chosen because below 15 °C, the methyl red in DMF forms a gel. The graphene and methyl-red inks were mixed with an optimum 2:1 ratio. The mixed ink was then placed on a bath sonicator at ambient conditions for 1 h to make a uniform dispersion solution of graphene flakes and methyl-red. The sensor electrical resistance changed from 11 MΩ to 0.4 MΩ towards the relative humidity content from 5% to 95%. The proposed humidity sensor showed 96.36% resistive and 2869500% capacitive sensitivity against humidity. The response and recovery time of the sensor was respectively equal to 0.25 s and 0.35 s. Furthermore, it had negligible cross sensitivity from other constituents in air because of M-R addition in the graphene.

Pang et al. [51] realized a porous material, 1.5 µm thick, by means of chemical vapor deposition, growing graphene flakes on a “model” nickel foam. The metallic skeleton was then dissolved with HCl. For performance improvement of Graphene Oxide sensors in moisture monitoring, the GO network was modified with poly (3,4-ethylenedioxythiophene) poly(styrenesulfonate) (PEDOT: PSS) and Ag colloids (AC). In this way, the sensor showed a response time of 31 s and a recovery time of 72 s. The relative resistance change (R−R_0_)/R_0_ reached a value of 1.10% at 12 RH% and increased up to 4.97% at 97 RH%. While the water molecules progressively accumulated in the graphene network, the resistance variation showed improvement. The field of applications of this technology ranged from healthcare to the detection of skin moisture and clinical respiration monitoring.

**Figure 12:**
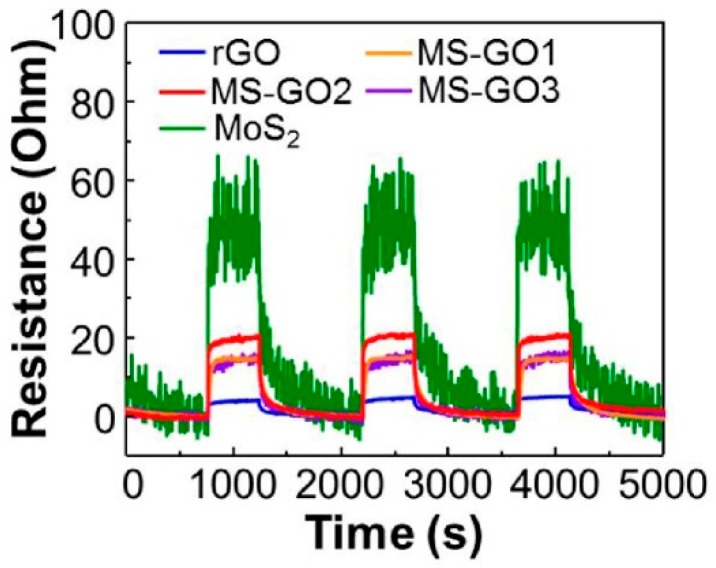
Sensing curves of rGO and MS-GO composites and of MoS2 when RH switched from 0% to 50%. Reproduced with permission from [52], published by Elsevier, 2018.

In Yun et al. [52], reduced graphene oxide and MoS_2_ hybrid composites were synthesized by the hydrothermal method and drop-cast on an SiO_2_ layer. The amount of GO was defined by the following molar ratio: GO/(NH_4_)_2_MoS_4_ = 5:1, 3:2, 1:5. These sensors were labelled as MS-GO1, MS-GO2, and MS-GO3. A p-n junction was formed between rGO and MoS_2_ due to van der Waals bonding and was responsible for short response and recovery times and a high selectivity, as well as a greater sensitivity and linearity for humidity sensing in comparison with pristine *p*- or *n*-type sensors. The responses of rGO, MS-GO1, MS-GO2, MS-GO3, and MoS_2_ sensors were equal to 3%, 15%, 21%, 14%, and 49%, respectively, at 50 RH% at room temperature (Figure 12). The response times of GO, MSG-O1, MS-GO2, MS-GO3, and MoS_2_ were 193.7, 59, 30, 116, and 17 s, and the recovery times of each sample were 418, 343, 253, 282, and 474 s, respectively. Although pristine MoS_2_ showed the highest response value, its recovery time was higher with respect to all other investigated samples, so MS-GO2 demonstrated the best overall sensing performances.

Finally, Park et al. [53] prepared sensors based on rGo/MoS_2_ at a molar ratio of 1: 1, 1: 5, and 1: 10 by simple ultrasonication. These samples were labelled RGMS 1, RGMS 5, and RGMS 10, respectively. RGMS 1; 5; 10 showed a sensor response of 10.5, 872.7, and 86.6% to 50 RH%, respectively. rGo/MoS_2_ in the proportion 1:5 showed the highest response to water vapor among the composites and a signal 220 times higher than that of pure rGo. In addition, the base resistance increased with the increasing molar ratio of MoS_2_. The responses of RGMS 5 to 50 ppm H_2_, 50 ppm CH_3_COCH_3_, 10 ppm NO_2_, and 1000 ppm NH_3_ at 27 °C and 1 V were 3.2%, 17.8%, 29.6%, and 47.9%, respectively.

### 2.3. Carbon Fibers

Carbon nanofiber-based gas sensors are much less popular with respect to those made with CNTs and graphene nanosheets [54].

Commercial carbon nanofibers, produced at temperatures above 1100°C from natural gas and sulfur in a floating nickel catalyst reactor, with a diameter between 20 and 80 nm and lengths of more than 30 μm, were first dispersed in 2-propanol and then, deposited over interdigitated silver electrodes on a PI substrate by means of a spray technique. The formed film was homogenous, with a thickness of hundreds of nanometers, except for the edges, which were thicker. Two kinds of nanofibers were tested: one with a graphitization of about 70% (GANF) and one with about 100% (GANFG) [55,56]. A linear increase of the resistance was observed for the GANF and GANFG sensors with the enhancing water vapor concentration in the range 5–100 RH%. However, a certain drift in the baseline was also observed, especially for relatively high humidity concentrations. Finally, GANFG displayed a better response to radiation (visible light and UV rays) and both types of nanofibers presented a better and faster response and were more repeatable to visible light than UV. It must be underlined that the effect of radiation cannot be ignored as an interference for sensing applications.

### 2.4. Carbon Films Produced by Physical Vapor Deposition (PVD)

**Figure 13:**
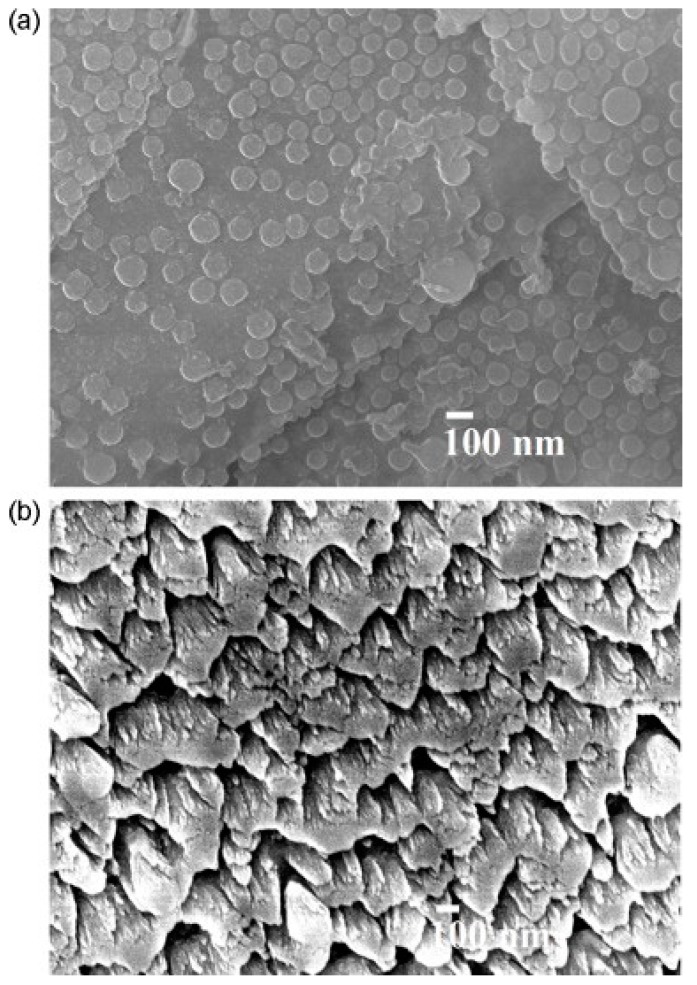
FESEM images of n-C films (thickness 550 nm) grown under CH_4_ (57.5 Pa). Nanoparticles with 80 nm size on the surfaces of carbon nanosheets can be observed (**a**). n-C films with 210 nm thickness grown in vacuum, nanohoneycomb structures are visible in (**b**). Reproduced with permission from [4], published by Elsevier, 2013.

**Figure 14:**
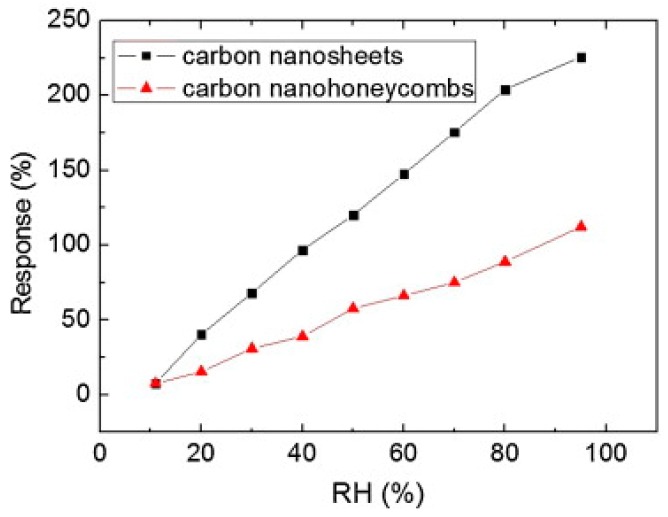
Humidity sensing properties of carbon nanosheets and nanohoneycombs-based sensors. Reproduced with permission from [4], published by Elsevier, 2013.

Films of nanostructured carbon (n-C) were grown from a graphite stick under methane and under vacuum by a hot filament physical vapor deposition (HFPVD) method on Si (100) substrates with an SiO_2_ buffer layer [4,57,58]. Four gold electrodes were then sputtered onto the n-C film. FESEM micrographs of n-C films showed the presence of nanoparticles (with an average size of about 80 nm), while for the sample grown in vacuum, vertically and well-organized nanohoneycomb structures were observed (Figure 13). The thickness of the two samples was 550 and 210 nm, respectively. The resistance of both n-C films changed almost linearly with RH (Figure 13). When RH rose from 11% to 95%, the resistances of the carbon nanosheets and nanohoneycombs-based sensors increased by 225% and 112%, respectively (Figure 14). 

The maximum hysteresis was about 3.57% and 6.83% under 50 RH% for the carbon nanosheets and nanohoneycombs-based films, respectively. The response time when the relative humidity level changed from 11 to 40 RH% was about 30 s, while the recovery time from 40 to 11 RH% was about 90 s for the carbon nanosheets film.

Amorphous carbon (a-C) capacitive films were deposited on n-silicon from a cold pressed graphite powder target by direct current magnetron sputtering at room temperature in [57]. The results showed that when the RH level changed from 11% to 95%, the junction capacitance showed an increase of ∼200% from ∼1000 pF at RH = 11% to ∼3200 pF at RH = 95%. When the RH amount was enhanced from 33 to 95%, the capacitance of the junction increased by 68% at a 1 kHz frequency. The response and recovery times were about 3 and 4 minutes, respectively.

In [58], hydrogenated amorphous carbon films (a-C:H) were made over printed circuit boards by physical deposition in vapor phase evaporation (PAVD) and plasma pulsed nitriding from graphite rods irradiated with an electron-beam. The aim of the latter treatment was to dilute the sample, to increase its resistance, and to modify the microstructure of the surface by interaction with the plasma. The sensors showed a relative impedance response equal to 0.99 when the RH value changed from 30% to 90%, when alimented with an AC tension of 1 V at 1 kHz.

N-doped carbon spheres (N-CSs)-poly (vinyl alcohol) (PVA) composites were deposited onto an epoxy resin/fiber glass board with gold interdigitated electrodes in [59]. First, acetylene gas (300 mL/min) was bubbled through acetonitrile heated at 80°C as a nitrogen source to produce N-CSs and then, was flowed in an argon atmosphere at 900°C for 10 minutes through a quartz tube [60]. The obtained powder was collected and purified by Soxhlet extraction from toluene for about 48 h, before being dried under vacuum for 48 h at room temperature [60]. The N-doped carbon spheres had a diameter of 181 ± 13 nm. The composite sensors were prepared by dispersing N-CSs in PVA matrix (molecular weight of 1.3 × 10^5^ amu). The dispersion was prepared by adding 6 mg·mL^−1^ hexadecyltrimethylammonium bromide (CTAB) and 4 mg·mL^−1^ of N-CSs to water [59]. This solution was ultrasonicated in an ultrasonic bath at room temperature for 30 min, followed by ultrasonication for 60 min at 0 °C. This dispersion was then left for four days at 10 °C, to let the excess surfactant precipitate in the form of hydrated crystals. After four days, 50% of the total volume of the supernatant free of hydrated crystals was removed and mixed with 6 mg·mL^−1^ of PVA in water. The proportion was chosen to obtain a final N-CSs weight content in PVA of 87%. Finally, sensors were prepared from the N-CSs/PVA—CTAB by dropping a 20, 30, and 35 μL dispersion on top of two types of gold interdigitated electrodes (IDEs) with a gap of 0.1 and 0.3 mm. In the RH range from 9% to 97%, the sensitivity of the sensor with a 0.1 mm gap was 17.1 1/RH%, while the sensitivity of the sensor with a 0.3 mm gap was 8.7 1/RH%. The response and recovery times were equal to 19 s and 178 s, respectively, for the IDEs with a 0.1 mm gap. They were then equal to 8 s and 142 s, respectively, when the IDEs had a gap of 0.3 mm.

An interesting self-powered humidity sensor is described in [61], where an electrical potential of tens of millivolts can be induced by the adsorption of water molecules on a piece of porous carbon film (PCF) with two sides functionalized with different functional groups deposited onto an alumina substrate. PCF was functionalized by simply exposing one half of the PCF to air plasma for one minute while covering the other half with a polyethylene film. This treatment allocates the plasma treated region a higher content of oxygen-containing functional groups, mainly –COOH, resulting in an improved hydrophilicity. The results showed that the water molecules adsorbed on the porous carbon film facilitate the release of protons from –COOH groups. Then, these protons can be freely transported through a bridge formed from adsorbed water molecules in porous carbon, while the –COO^−^ groups on the carbon surface remain negative. The produced voltage is due to two consecutive processes: proton release and proton transport. Both steps are restricted by the amount of adsorbed water molecules. Ab initio molecular dynamics modeling revealed that a small amount of water molecules could facilitate the proton release from the –COOH groups, forming an effective water bridge for proton transport. These results explain why the voltage remains at zero at low humidity values, starts to rise at 94 RH%, and reaches a maximum when the humidity is close to saturation. Thus, a possible application for these sensors could be as dew point sensors.

### 2.5. Carbon Black and Biochar

Carbon black-filled polymers have been studied with the aim of developing sensitive and cheap sensors. Carbon black (CB) impairs electrical conductivity to the film which, at high RH levels, swells and increases the resistance of the film. In [62], films of CB and the Fe[(Htrz)_2_(trz)]BF_4_ complex (where *Htrz* = 1,2,4-1H-triazole and *trz* is the deprotonated triazole ligand) were screen-printed. The nanoparticles were realized by a reverse micelle method and had an average diameter of 45 nm. They were dispersed in a commercial carbon black-epoxy ink (ElectroScience Laboratory, ESL RS12113) and screen-printed on an alumina substrate. While sensors based on carbon-black only tend to reach saturation at 50 RH% levels, this result was not observed with the sensors based on carbon-black and Fe(II) compound hybrids. In addition, the latter showed a six-time higher sensitivity compared to the pristine ones. 

In [63], carbon quantum dots (CQDs) films were obtained from two identical graphite rods (99.99%, Alfa Aesar, 13 cm in length and 0.6 cm in diameter) vertically inserted in an ultrapure water solution 3 cm above the liquid surface. Both rods were respectively the cathode and the anode of an electrolytic cell and were separated by 7.5 cm. Then, a static potential of 50 V was imposed on the two electrodes by a direct current power supply for 10 days under thorough stirring. Finally, the large graphite particles were removed from the CQDs solution by high speed centrifugation (22,000 rpm). The concentration of the CQDs in aqueous solution was determined to be about 1.2 mg·mL^−1^. Finally, the CQDs films were manufactured by dropping 2 mL of CQDs aqueous solution on a piece of glass sheet (1 cm × 2 cm), which was then dried at 25 °C. The films were 3 μm in thickness.

In the same work, the authors also used 500 mg of candle soot, which was mixed with 100 mL of 5 M HNO_3_. As a result, candle soot nanoparticles were functionalized with oxygen-containing groups by refluxing the fresh candle soot in nitric acid at 100 °C for 24 h. After cooling down to room temperature, the fluorescent carbon nanoparticles were collected by centrifugation (10,000 rpm for 10 min). The films based on candle soot were prepared in the same way as the CQDs.

High-resolution TEM observations evidenced that the resulting CQDs had a uniform particle size distribution ranging from 4 nm to 8 nm. 

At low RH levels, only a few high-energy electrons can accomplish the leap migration process between neighboring CQDs. Thus, the conductivity of the solid CQDs films in a dry environment is very low. However, at high RH values, the oxygen-contained groups on the surface of the neighboring CQDs can form hydrogen bonds with water molecules, significantly favoring the migration of electrons and then, increasing the conductivity. Because electron migration between adjacent CQDs through hydrogen bonds is much easier than that through the jumping mode, the conductivity of the CQDs film sharply increases with the humidity of the surrounding environment. 

**Figure 15:**
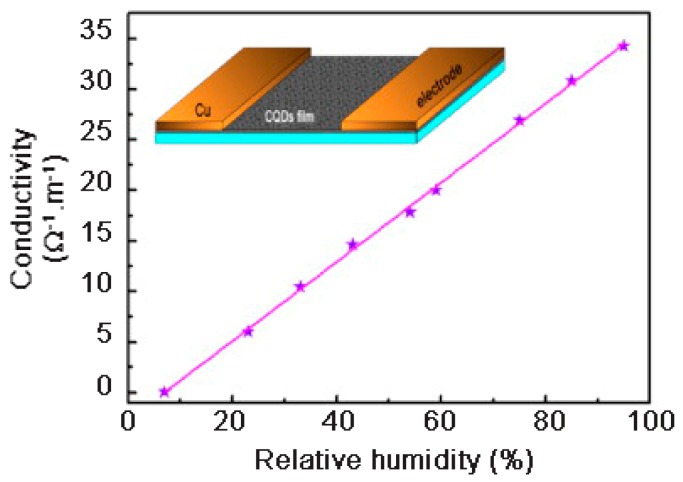
The conductivity of the CQDs film as a function of the relative humidity at room temperature. Reproduced with permission from [63], published by Elsevier, 2013.

The CQDs films proved to be highly sensitive to water vapor and showed a linear dependence of the conductivity with the relative humidity (Figure 15). The oxygen-containing groups on the surface were considered to be of paramount importance for humidity detection by CQDs. The conductivity of the CQDs film in a dry environment (below 7 RH%) was close to zero, whereas when the CQDs film was placed under 95 RH%, it showed a conductivity increase up to ~35 Ω^−1^·m^−1^. In addition, the response and recovery times were measured between 7 and 43 RH% at room temperature and were equal to ~25 s and ~60 s, respectively. Beside the fast response time, the CQDs-based humidity sensor also showed an excellent stability, even for a long-term test [63].

A polyvinylpyrrolidone-grafted carbon black (CB-PVP) composite was prepared by a free radical polymerization reaction [64]. Hydrophilic polyvinylpyrrolidone (PVP) was grafted onto the CB particles (BP-2000 CB from Cabot Limited Corporation, USA) to obtain a water-dispersible conductive material. BP-2000 CB material was selected as the conductive material because of its high specific surface area and small particle size (15–20 nm in diameter). The grafting of PVP onto CB was performed as follows: 1.3 g of CB, 5.4 g of N-vinypyrrolidone (NVP), 0.03 g of 2,2′-azobisisobutyronitrile (AIBN), and 18 mL of tetrahydrofuran (THF) were added to a 100 mL flask and heated at 60 °C for 6 h. Then, the reaction mixture was centrifuged several times to remove the ungrafted polymer and AIBN. The obtained solids were further purified by Soxhlet extraction with THF for 48 h and dried under vacuum at 40 °C. The content of PVP in CB-PVP particles was about 5.3 wt%.

**Figure 16:**
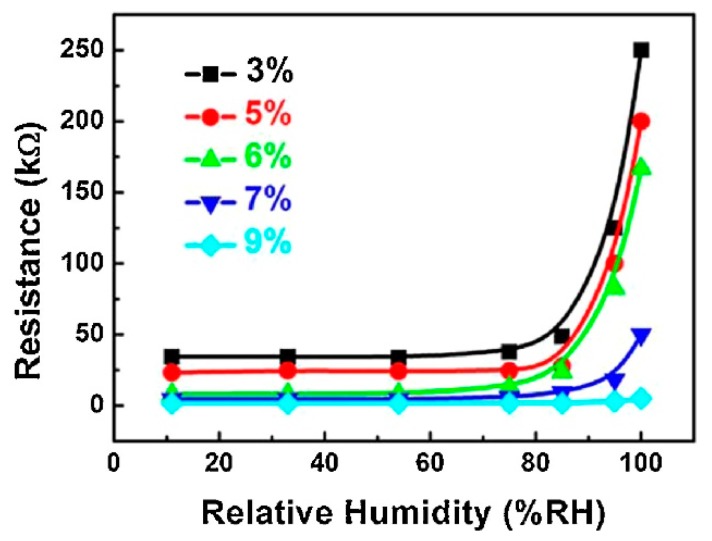
The resistances of CB-PVP/PVA composite films with different contents of CB as a function of RH%. Reproduced with permission from [64], published by Elsevier, 2014.

For the sensors’ preparation, an appropriate amount of CB-PVP and PVA was added into a conical flask with distilled water and then heated at 75 °C and kept under stirring for 2 h to form a stable suspension. After cooling to room temperature, the suspensions (with 3, 5, 6, 7, and 9 wt% of CB-PVP in PVA) were cast onto clean ceramic substrates (10 mm × 8 mm × 0.8 mm) with a screen-printed interdigitated array of Ag–Pd electrodes. The thickness of the film was about 30 μm. A six-hour thermal annealing treatment at 100 °C was performed to enhance the stability of CB-PVP/PVA composite films. The resistance values of composite films were small under low RH levels and increased notably at a certain RH. When the RH value is low, the PVA film adsorbs a small quantity of water, and the space between the CB particles is small enough to form conductive channels. With the increase of RH, the polymers swell and the space between CB particles increases slowly, as does the resistance of composite films. When the RH level is above 85%, rapid swelling of the composite film leads to a sharp decrease of CB concentration, so the resistances of composite membranes increase sharply (Figure 16).

To the best of our knowledge, only a few studies are dealing with the use of biochar materials for humidity monitoring at room temperature [21,65,66].

In Afify et al. [65], pyrolyzed bamboo screen-printed thick-films were used as novel humidity sensors. Split bamboo culms were cut into small pieces (~1–5 mm) and cleaned with distilled water prior to drying in an oven at 105 ± 5 °C for 48 h. The dried bamboo pieces were then pyrolyzed in a quartz reactor at 800 °C for 1 h under an inert atmosphere of argon gas. The carbonized bamboo (CB) pieces were first manually ground in an agate mortar with an agate pestle, and then by attrition milling in distilled water for 1 h. Subsequently, the powder was dispersed in an organic solvent (ethylene glycol monobutyal ether, Emflow), which provides the appropriate rheological properties to the paste. Once screen-printed onto alumina substrates with platinum interdigitated electrodes, the sensors were dried in air at room temperature and heat-treated at 300 °C in air for 1 h, to remove organic residues from the solvent. CB sensors showed a significant response towards RH at room temperature with a cut-off RH at about 10%. The sensors’ resistance started from about 937 kOhm at 0% RH and decreased to 89 kOhm at 95% RH for CB, while the maximum hysteresis was 28.9% at about 33 RH%. The response and the recovery times were quite fast (about 2 min), with recovery times always being shorter with respect to response times. Short response times may be due to the porosity of the film, which gives great accessibility for water molecules’ adsorption. Fast recovery times indicate that the process of physisorption was probably the major reason for binding water molecules to the sensing materials. However, the adhesion onto alumina substrates of these sensing films was minimal and they could be easily damaged when testing. Thus, their use in commercial products was precluded.

**Figure 17:**
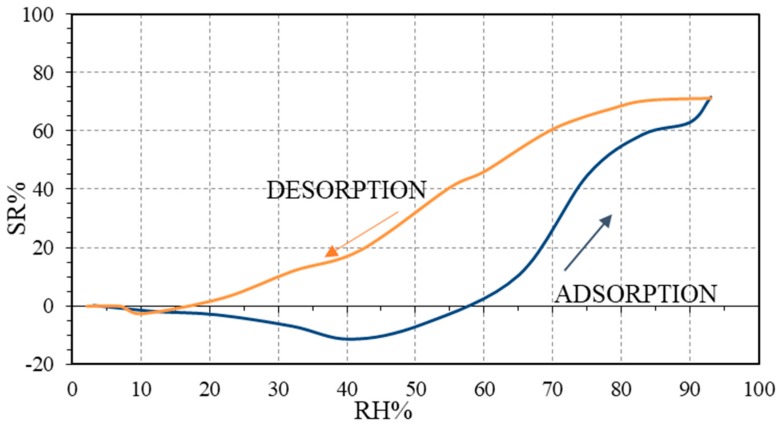
Sensor response (SR%) towards relative humidity for SWP700 biochar with 10 wt% of PVP. Reproduced with permission from [21], published by MDPI, 2017.

In Ziegler et al. [21], SWP700 (pyrolyzed mixed softwood pellets) and OSR700 (oil seed rape) commercial biochars (from UK Biochar Research Centre) were used as humidity sensitive materials. The sensors, screen-printed onto alumina substrates with platinum interdigitated electrodes, exhibited a *p*-type behavior at low humidity values (between 5–25 RH% for SWP700 and in the range 5–40 RH% for OSR sensor). Specifically, for the OSR700 film, the initial impedance under dry air was equal to 163.9 kΩ, and the final impedance under 99 RH% decreased to 9 kΩ, leading to a sensor response equal to 94.5%, together with a maximum hysteresis of 52% under 59 of RH% (Figure 17). For the SWP 700 film, due to the highest surface area and porosity compared to the previous biochar, the impedance dropped from 9.5 MΩ in a dry condition to 222 kΩ in a humid environment, with a sensor response of 97.6%. This material exhibited a lower hysteresis, with a maximum value for this parameter of 23% under 55 RH%. Using SWP700, the biochar most sensitive towards humidity, sensors with different amounts of PVP were produced (10 and 20 wt% with respect to biochar). The SWP700 sensor with 10 wt% PVP seemed to represent a satisfying compromise between sensitivity towards water vapor and adhesion of the film onto the substrate (Figure 17). 

No interferences were detected towards 0.5 ppm of ozone, 100 ppm of methane, and 500 and 600 ppm (under dry and humid (40 RH%) air) of carbon dioxide. Only a slight increase in impedance of 2.6% was evidenced under 50 ppm of ammonia at room temperature.

**Figure 18:**
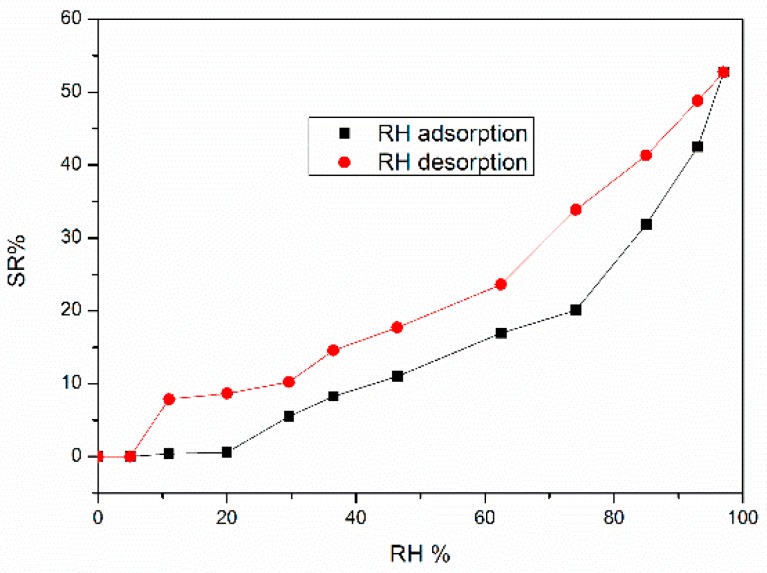
Sensor response (SR%) towards relative humidity for a coffee ground biochar sensor during adsorption and desorption cycles. Reproduced with permission from [66], published by MDPI, 2019.

Finally, in Jagdale et al. [66], waste brewed coffee powder (WBCP) was first washed with water, centrifuged, and then filtered. Afterward, the WBCP powder was then dried in an oven at 90°C for 10 h. The pyrolysis of the material was performed at 700 °C for 1 h in nitrogen atmosphere (120 mL/min) with 30 min dwells, respectively, at 250 °C and 400 °C. After the pyrolysis step, the material was manually ground, leading to coffee ground biochar, before being screen-printed onto alumina substrates with platinum interdigitated electrodes. The sensors behaved as *n*-type semiconductors (Figure 18), with the conductivity increasing with the humidity level: the sensor response SR% started at around 20 RH% and reached 51% under humid atmospheres (98% of RH). The impedance decreased from 25.2 MΩ in dry conditions to 12.3 MΩ, in humid conditions. The high initial impedance value was probably due to the limited thickness of the sensing film. The maximum hysteresis was equal to 15% under 74 RH%, confirming that the kinetics of desorption is comparable with respect to the adsorption one. The response and recovery times under 50% of RH were determined and were respectively equal to 4.5 and 1 min. Additionally, negligible interferences were detected towards carbon dioxide 500 ppm, ozone 200 ppb, nitrogen dioxide 200 ppb, and ammonia 50 ppm with this sensing material. 

The main features of the different sensors described in this paper are listed in Table 2.

## 3. Interaction with Water Molecules

Water molecules can adsorb on the surface of carbon films due to a weak binding of water hydrogens to surface carbon atoms [4]. In addition, the sensing performances are strongly influenced by the presence of defects in carbon particles, which could create favorable adsorption sites for water molecules [4]. At low RH levels, a few water vapor molecules chemically adsorb on the carbon grain surfaces. Pati et al. [67] suggested a possible charge transfer between the adsorbate and the carbon film. On carbon nanotubes, H_2_O molecule adsorption yields a charge transfer to CNTs of 0.033–0.035 electron per water molecule, as demonstrated by theoretical studies and experimental results [4]. These charge transfer effects determine an increase in the resistance of the CNTs-based gas sensors [68,69,70,71]. In fact, this probably leads to electrons transfer to the valence band and to an increase of the impedance as the conductivity of *p*-type semiconductors is determined by holes scattering through the material. Typically, carbon-based materials contain numerous oxygen derivatives on their surface. These functional groups can improve the carbon surface hydrophilicity and increase water vapor molecules adsorption [72]. When these adsorbed water molecules donate electrons to the valence band of the carbon material, the number of holes decreases and the separation in energy between the Fermi level and valence band increases [73,74], decreasing the conductivity of the *p*-type semiconductor. The higher the RH level, the more water molecules are adsorbed, and more electrons are transferred, lowering the holes’ concentration and leading to an increase of the resistance value of samples by raising RH values [4]. An increase in sensitivity towards H_2_O was also observed upon the functionalization of pristine CNTs with different molecules [75].

On the contrary, for higher RH levels, when more water molecules are adsorbed on the surface, a liquid-like multilayer of hydrogen-bonded water molecules will form at room temperature [76]. In addition, these physisorbed water molecules can condense into pores with a size in the range of 1–250 nm. Since the formation of clusters of H_2_O and the hydration of H^+^ into H_3_O^+^ are thermodynamically favored in liquid water, H^+^ are the dominant charge carriers in the water adsorbed in the mesopores. As the amount of H^+^ enhances when increasing the moisture content, H^+^ can move freely in liquid water, leading to a decrease of grain surface impedance with heightening RH values [76].

According to Ref. [77], at high humidity amounts, the conduction is well-described by the Grotthuss mechanism. It might be that the proton mobility correlates with H-bond cleavage rather than with its formation, since it enhances when decreasing the H bond content in water, i.e., at higher temperatures and pressures. The proton mobility is an incoherent proton hopping phenomenon. The actual proton motion is much faster compared to the solvent reorganization. The rate-limiting step involves the cleavage of a single H having an activation energy of about 2.6 kCal/mol. During the elementary transfer step itself, the donor and acceptor H_2_O with their solvation shells present equivalent structures. Since the rate-limiting step involves the cleavage of a H bond, but not one in the first solvation shell, H-bond cleavage in the second solvation shell is the rate-limiting step. This leads to isomerization of the H_9_O_4_^+^ cation into H_5_O_2_^+^ The proof of this phenomenon is more likely to come from computational chemistry than from any single experiment.

When increasing the concentration of the reducing gas to which the nanotubes are exposed, the Fermi level may shift and the CNTs could turn from *p*-type into *n*-type materials: for instance, in [71], the response of the composite was *p*-type at low concentrations of NH_3_ under low RH values and switched to *n*-type in higher humidity conditions. This behavior was attributed to a hole compensation effect by water molecules [71]. 

Zhang et al. showed that the resistance reduction of the single-walled carbon nanotube (SWCNTs) networks is caused by the SWCNTs, while the resistance increase is due to the inter-tube junctions [63]. Then, the overall resistive humidity response of the SWCNT network is the result of a competition between the SWCNT resistance changes and intertube junction resistances’ variations. In fact, intertube junctions play a crucial role in limiting the SWCNT network conductance due to the suppression of intertube carrier hopping caused by water molecules. Covalent modifications to the SWCNTs were shown to be able to raise the humidity detection dynamic ranges by increasing the resistance of the SWCNTs film. These modifications also enhanced the sensitivities of as-grown SWCNT networks to humidity by increasing their hydrophilicity.

## 4. Conclusions and Perspectives

Flexible sensors with a high sensitivity, excellent flexibility, acceptable stretchability, and good stability, can be mounted on the human body or clothing to provide the long-term detection of human activities and physiological information [12]. Thus, future research aims at developing more sensitive, selective, and stable humidity sensors able to withstand harsh environments in a wide range of temperatures and in dynamic operating modes [1]. Though important progress in flexible sensors based on carbonaceous materials has been achieved, some key issues, including the preparation processes of carbon materials, the fabrication processes, and the performance of flexible sensors, still need to be further investigated for practical applications [12]. Mass and cost-effective production of carbon materials with a high quality is a fundamental prerequisite for these targeted applications. CNT powders and GO sheets have been embedded in mass production, but the manufacturing processes can also introduce many defects, significantly affecting their humidity sensing properties. A monolayer and few-layers of graphene with a high quality are mandatory for fabricating high-performance devices. However, the current high-cost preparation methods limit their use in view of industrial applications. In addition, the properties of macroscopic assemblies are clearly lower than those of individual CNTs or graphene [12]. 

Moreover, bio-materials (cotton fibers) with macro-scale architectures (woven structures) are available. After a simple carbonization treatment (pyrolysis), they can be used as active materials of high-performance flexible sensors [14,15]. As shown, from the pyrolysis of bio-materials, it is possible to obtain additionally encouraging carbon sensing materials for fabricating high-performance flexible sensors. 

Gas sensors are mainly produced in two ways: solution casting and CVD deposition. Solution casting processes include simple casting, dropping-drying, spin-coating, and dip-coating. The suspensions for film casting on certain substrates can be simply produced by direct dispersion of the nanocarbon components in solvents. These simple and cost-effective methods can be easily scaled-up for industrial applications [1]. In CVD methods, nanocarbon (CNTs and graphene sheets) layers can be directly grown on substrates. Solution casting methods are more flexible when preparing composite materials, while thin-films manufactured by CVD are more uniform and homogeneous [1]. It is expected that composites of nanocarbons and nanoparticles of polymers and carbonaceous materials should be the main topic of this research direction, due to the possibility to obtain easily flexible films.

Finally, the integration of flexible sensors with energy conversion and storage devices is required in view of the practical applications of wearable electronics. However, these self-powered sensors still require external power for operating the measurement systems and reading the signals [12]. Therefore, the integration of self-powered components, energy storage devices (such as supercapacitors or batteries), and flexible sensors is of paramount importance for manufacturing wearable systems [12].

## Figures and Tables

**Table 1:** Sensitivity of MWCNTs/PI composites. Reproduced with permission from [33], published by Elsevier, 2010.

CNT Concentration (wt%/PI)	p-MWCNTs/PI Sensitivity (1/RH%)	MWCNTs/PI Sensitivity (1/RH%)
0.1	0.00127	0.00092
0.2	0.00149	0.00103
0.3	0.00305	0.00182
0.4	0.00466	0.00218

**Table 2:** Main features of humidity sensors based on carbonaceous materials. n.d.–not determined.

Sample	Sensor Response	Response Time	Recovery Time	Ref.
MWCNTs in a PAA matrix in a ratio 1:4	930 Ω increase of the resistance when RH changed from 30% to 90%	680 s when RH increased from 50% to 90%	380 s when RH decreased from 90% to 50%	[5]
MWCNTs CVD grown on quartz substrates	~267% increase of resistance under 100 RH%	2–3 min	Few hours	[29]
3 wt% MWCNTs in a PI matrix	~10% increase of resistance when RH changed from 30% to 90%	< 5 s when RH increased from 30% to 90%	n.d.	[32]
0.4 wt% MWCNTs in a PI matrix	~40% increase of resistance when RH changed from 10% to 90%	n.d.	n.d.	[33]
9 wt% MWCNTs in a HEC matrix	~300% increase of resistance change ratio when RH changed from 23% to 80%	~1500 s when RH increased from 0% to 90%	n.d.	[35]
MWCNTs in a PVP matrix in a ratio 1:9	4000% current ratio at 94 RH%	15 s	1.8 s	[36]
SWCNTs/PVA filaments in a molar ratio 1:5	Resistance increase of 24 times	40 s	n.d.	[37]
MnO_2_-coated CNT yarn	Resistance variation of 65% under 90% of RH	20 s	30 s	[38]
Defect graphene on alumina substrate with gold electrodes	Relative conductance ratio of 3.33 when RH changed from 3% to 30%	n.d.	~150 s when RH% decreased from 30% to 3%	[39]
Spin-coated GO films on PET substrates + laser treatment, 0.2 W	~20 kΩ increase of resistance when RH changed from 11% to 95%	3 s when RH% increased from 11% to 95%	10 s when RH% decreased from 95% to 11%	[42]
GO foam	~37% decrease of the current when RH increased from 0% to 85.9%	89 ms when RH% increased from 0% to 85.9%	189 ms when RH% decreased from 85.9% to 0%	[44]
rGO on PDMS substrate	~2750% increase of the capacitance when RH increased from 20% to 90%	n.d.	n.d.	[46]
GO on polyethylene naphthlate by by drop casting and spray coating methods	The sensor response was normalized	< 100 ms	< 100 ms	[47]
Single layer of graphene on SiO_2_ by CVD	Relative resistance variation of 1.2% in the range 8–85 RH%	0.6 s	0.4 s	[48]
Tin dioxide/reduced graphene oxide (RGO) nanocomposite film	Capacitance from 246.53 pF at 11% of RH to 138,267 pF at 97%	102 s	Several s	[49]
Graphene/methyl-red composite	SR % 96.36 in terms of reistance and 2869500% in terms of capacitance	0.251 s	0.35 s	[50]
GO modified with poly (3,4-ethylenedioxythiophene) poly(styrenesulfonate) (PEDOT: PSS) and Ag colloids	4.97% under 97 RH%	31 s	72 s	[51]
Reduced graphene oxide and MoS2 hybrid composites were synthesized by hydrothermal method and drop-cast	21% under 50 RH%	30 s	253 s	[52]
rGo/MoS_2_ composites by sonication	872.7% under 50 RH%	6.3 s	30.8 s	[53]
C nanofibers sprayed on a PI substrate	~16% increase of relative resistance ratio when RH increased from 5% to 100%	n.d.	n.d.	[56]
C nanosheets produced by physical vapor deposition	Resistance increase of 225% under 95 RH%	30 s when RH% increased from 11% to 40%	90 s when RH% decreased from 40% to 11%	[4]
Amorphous carbon film by DC magnetron sputtering	~200% increase of capacitance when RH increased from 11% to 95%	3 minutes when RH% increased from 33% to 95%	4 minutes when RH% decreased from 95% to 33%	[57]
Hydrogenated amorphous carbon (a-C:H) film	Resistance decrease of 97.3% under 80 RH%	n.d.	n.d.	[58]
N-doped carbon spheres (N-CSs- PVA) by drop coating (0.1 mm gap IDEs)	Conductance increased by 4 order of magnitude in th eRH range 9–97%	19 s	178 s	[59]
Screen-printed commercial composite ink (ESL RS12113) made of epoxy resin and carbon powder	Resistance increase of 4.8% under 80 RH%	n.d.	n.d.	[62]
Carbon quantum dots film made by electrochemical ablation of graphite	Resistivity decrease of 48% under 90 RH%	25 s when RH% increases from 7% to 43%	60 s when RH% decreases from 43% to 7%	[63]
Pyrolyzed bamboo	Resistance decrease of 91% under 95% RH	2 min	2 min	[65]
Pyrolyzed mixed softwood pellets	Impedance decrease of 97.7% under 97.5% RH	1 min	1 min	[21]
Oil seed rape	Impedance decrease of 94.5% under 99% RH	50 s	70 s	[21]
Coffee ground biochar	Impedance decrease of 51% under 98% RH	4.5 min	1 min	[66]
